# Enhancing vertically stacked skin display compactness via discrete layer preparation

**DOI:** 10.1038/s41377-024-01553-8

**Published:** 2024-08-24

**Authors:** Yichen Cai, Xiangyu Hou, Wei Chen

**Affiliations:** 1 Department of Chemistry, National University of Singapore, Singapore, 117543 Singapore; 2 Department of Physics, National University of Singapore, Singapore, 117542 Singapore; 3 Joint School of National University of Singapore and Tianjin University, International Campus of Tianjin University, Binhai New City, Fuzhou, 350207 China; 4 National University of Singapore (Suzhou) Research Institute, Suzhou, 215123 China

**Keywords:** Applied optics, Electronics, photonics and device physics

## Abstract

The discrete preparation of functional layers followed by lamination for all-organic active-matrix organic light-emitting diodes enables an ultrahigh aperture ratio and reliable conformability, promising significant potential for next-generation skin-like displays.

Over the past few decades, wearable electronics have advanced rapidly, driven by breakthroughs in microelectronics, battery technology, and wireless communications^1–3^. Among the various wearable display technologies, skin-like displays have emerged as a promising interface for human-machine interaction, particularly in health monitoring systems^4–6^. Key considerations for skin-like displays include flexibility and biocompatibility. Recent strides in active-matrix organic light-emitting diodes (AMOLEDs) have paved the way for seamless, low-power, and highly durable displays^7–9^.

AMOLEDs feature individual pixels controlled by addressable thin-film transistors (TFTs)^10^. However, integrating OLEDs and TFTs poses challenges, such as aperture ratio limitations compared to passive structures where OLEDs are directly driven by top and bottom electrodes. The parallel arrangement of OLEDs and TFTs reduces the aperture ratio since TFTs do not emit light^11^. Vertical stacking of OLEDs and TFTs has been proposed as a solution^12,13^, but it introduces complexities in layer-by-layer processes, materials compatibility, and defect spread. Developing a robust and commercially viable vertical stacking AMOLED process remains a significant challenge.

In a newly published research work in *Light: Science & Applications*, the research team led by Qingxin Tang, Xiaoli Zhao, Yongjun Dong, and Yichun Liu from Northeast Normal University, China, has proposed an innovative “discrete preparation-multilayer lamination” integration strategy to address the fabrication challenges of vertical stacking all-organic AMOLEDs^14^. In this approach, active layers—including OLEDs, organic thin-film transistors (OTFTs), and interconnect layers—are initially prepared separately and aligned laminated in the final steps. It effectively addresses challenges associated with conventional layer-by-layer fabrication, such as chemical and physical damage, and reduces the need for protective and pixel-definition layers. The significance of this strategy lies in the impressive aperture ratio of 83%, as well as excellent mechanical conformability and functional reliability.

In integrated display systems, every functional component plays a crucial role. To enhance the performance of vertical stacking display systems, the authors began with the material selection and processing of active layers. At the heart of the interconnect layer, which bridges top-emitting OLED (TE-OLED) and OTFTs, are requirements for excellent electrical conductivity, adhesion, and mechanical compatibility. For optimal lamination reliability, poly(styreneethylene-butylene-styrene) (SEBS) was chosen as the substrate, valued for its inherent dielectric property and viscoelasticity. Through precise photolithography and plasma etching, vias in the SEBS substrate were formed and subsequently filled with highly fluid conductive polymer PEDOT:PSS. In addition, an Au layer was deposited on the other side for OTFT connecting.

For the OLED layer, the performance of TE-OLED is the precondition for a high aperture ratio and superior electroluminescence. The authors utilized modified PEDOT:PSS as the anode and a hybrid metal film as the cathode to enhance electrical properties, transparency, and flexibility. Figure 3a of ref. ^14^ illustrates the energy-level matching OLED structure, delivering an impressive power efficiency of 11.56 lm W^−1^. Notably, this all-organic design retains 80% of its performance even at a 2-mm bend radius.

For the OTFT layer, ensuring stable operation to supply continuous current to OLEDs is critical. The OTFT array of bottom-gate, top-contact configuration was fabricated through a photolithography process with the active layer, indacenodithiophene-benzothiadiazole (IDT-BT). This configuration reaches a remarkable mobility of 1.36 cm^2^ V^−1^ s^−1^ and exhibits good uniformity and flexibility, laying a solid foundation for subsequent system integration.

Under the multilayer electrode pre-design, TE-OLED/interconnect/OTFT layers are precisely aligned and tightly stacked, as illustrated in fig. 5a of ref. ^14^. In this demonstration, each pixel comprises one OTFT and one OLED (1T-1D). The laminated AMOLED array not only preserves the excellent properties of individual layers but also benefits from a reliable interconnection, facilitating seamless integration for on-skin displays (Figure 5h of ref. ^14^). Furthermore, controlled activation of multiple pixels within the AMOLED array enables displaying given patterns.

This work marks a significant advancement in the development of vertically stacked skin-like AMOLEDs. This breakthrough demonstrates excellent mechanical flexibility, high display stability, and uniformity for the future development of wearable electronic devices. Multifunctional OLEDs and other flexible substrates can be stacked in this manner to achieve more commercial applications. For instance, OLEDs with multiple stimuli-responsive layers that exhibit different light-emitting patterns and colors in response to photo or electro-stimuli can be combined with OTFTs for next-generation flexible anticounterfeiting technologies^15^. This innovation holds promise for applications such as anticounterfeiting labels and the identification and verification of wearable devices.
